# Burkitt-like lymphoma of the brain mimicking an intraventricular colloid cyst

**DOI:** 10.1590/0100-3984.2016.0065

**Published:** 2017

**Authors:** Rodolfo Mendes Queiroz, Lucas Giansante Abud, Thiago Giansante Abud, Cecília Hissae Miyake, Antonio Carlos dos Santos

**Affiliations:** 1 Documenta - Hospital São Francisco, Ribeirão Preto, SP, Brazil; 2 Hospital Israelita Albert Einstein, São Paulo, SP, Brazil; 3 Hospital das Clínicas da Faculdade de Medicina de Ribeirão Preto da Universidade de São Paulo (HCFMRP-USP), Ribeirão Preto, SP, Brazil

**Figure 1:**
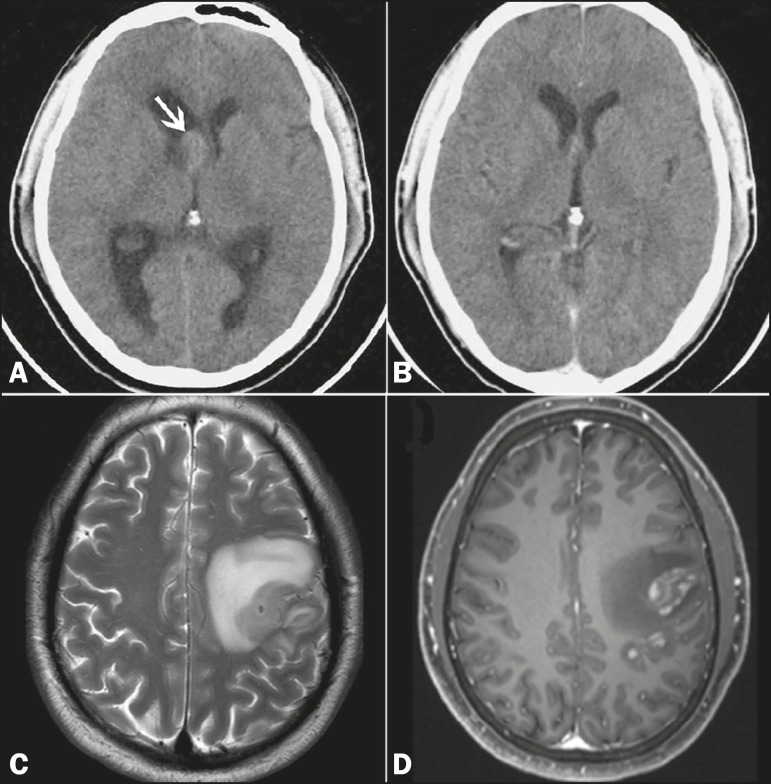
**A:** Non-contrast-enhanced CT scan of the brain, showing well-delineated, discretely hyperdense intraventricular nodule to the right of the foramen of Monro (arrow), promoting slight dilation of the lateral ventricles (obstructive hydrocephalus). **B:** Follow-up CT of the brain, obtained one month later, showing no such nodule. **C,D:** MRI of the brain after episodes of seizures, T2-weighted sequence (**C**) and paramagnetic contrast-enhanced T1-weighted sequence (**D**), showing an intra-axial frontoparietal mass in the left cerebral hemisphere, with intense perilesional vasogenic edema and heterogeneous enhancement.

*Dear Editor*,

A 32-year-old male sought treatment, complaining of headache. Computed tomography (CT) of the brain revealed hyperdense intraventricular nodule to the right of the foramen of Monro, highly suggestive of a colloid cyst (Figure 1A). The patient was using dexamethasone as pain therapy. In a CT scan of the brain obtained one month later, no nodules were observed (Figure 1B). Cervical and thoracoabdominal CT scans also showed no abnormalities. At two months, the patient presented with convulsions. Magnetic resonance imaging (MRI) of the brain showed a cerebral mass (Figures 1C and 1D). Histopathological and immunohistochemical analysis of a biopsy sample revealed Burkitt-like lymphoma, which is one of the non-Hodgkin lymphomas. Ancillary examinations ruled out systemic disease and viral infection.

Lymphomas are designated primary when they originate at and are confined to a given site^(1-3)^. Primary central nervous system (CNS) lymphomas account for up to 6% of brain neoplasms and 1-6% of extranodal lymphomas; approximately 90% of primary CNS lymphomas are non-Hodgkin lymphomas of the diffuse large B-cell subtype^(1-6)^. The incidence of CNS lymphoma is higher in the presence of certain immunodeficiencies, especially human immunodeficiency virus (HIV) infection^(2)^. Among immunocompetent individuals, the prevalence of CNS lymphoma is highest (60-67%) in men 45-75 years of age. In that group, CNS lymphomas present as a single homogeneous mass (in 62%), often in the supratentorial compartment (in 83%) and notably in the deep white matter (in 57%). The corpus callosum and regions surrounding the ventricles are typically affected. Perilesional edema is also common, being seen in 77-90% of cases^(1,3-6)^. On CT scans, CNS lymphomas are typically hyperdense, because they are hypercellular and have a high nucleus-cytoplasm ratio^(1,3)^. On MRI, they often demonstrate a hypointense or isointense signal in T1-weighted sequences and an isointense or hyperintense signal in T2-weighted sequences. After intravenous administration of contrast medium, they show homogeneous (90%) or, in rare cases, annular enhancement. They also exhibit signs of restricted water diffusion. Perfusion-weighted imaging shows less vascularization than that seen in other malignant brain tumors. On magnetic resonance spectroscopy, CNS lymphomas show elevated lipid and choline peaks, as well as a reduction in N-acetyl-aspartate levels^(1,3-5)^. The definitive diagnosis is made by biopsy^(1,2,4,6)^. Such lymphomas respond to chemotherapy and radiotherapy, the surgical option being used for tumor mass reduction^(1,3-5)^. Overall survival ranges from 15% to 80%, depending on the age of the patient, as well as on the characteristics and stage of the disease^(2,4)^.

The list of differential diagnoses of expansile CNS lesions in imaging studies is extensive, including glioma, acute ischemia, inflammatory processes, and infectious diseases^(1,3-5,7-11)^. When such lesions appear in an intraventricular location and are hyperdense on CT, they can be confused with colloid cysts, which are common at that site and exhibit similar density^(4)^.

Burkitt-like lymphomas are highly malignant, with cellular characteristics intermediate between those of diffuse non-Hodgkin large B-cell lymphoma and those of Burkitt lymphoma^(12-14)^. Burkitt-like lymphomas are typically associated with infection-HIV or the Epstein-Barr virus. They account for 2-3% of non-Hodgkin lymphomas in immunocompetent adults, being most common among the elderly^(12-14)^. Burkitt-like lymphomas can affect the brain, intestines, skin, ovaries, kidneys, liver, and bone marrow^(12)^. Chemotherapy is the most widely used treatment, although, even with treatment, survival is less than one year^(13,14)^.

The term "vanishing tumor" refers to a tumor that shows marked regression or disappears, with or without nonspecific therapy, and can recur or progress to new forms^(2,4,15,16)^. In the brain, lymphomas often occur after corticosteroid therapy, demyelinating diseases, or inflammatory disorders^(15,16)^.
